# Supplementary material for: COVID-19 pandemic impact on dentists in Latin America’s epicenter: São-Paulo, Brazil
Source: PLoS One. 2021 Aug 26;16(8):e0256092. doi: 10.1371/journal.pone.0256092 (PMC8389449; doi:10.1371/journal.pone.0256092)
Supplement: S2 Table — (original language: Brazilian Portuguese). (DOC) [file pone.0256092.s002.doc]

**S2 Table. Questionnaire (original language: Brazilian Portuguese)**

**DIMENSÃO 1 – CARACTERÍSTICAS GERAIS DA POPULAÇÃO ENTREVISTADA**

**Email: (Opcional)**

1. **Qual cidade você mora?**
2. **Qual cidade você trabalha?**
3. **Como você se indentifica?**

( ) Homem

( ) Mulher

( ) Outro

1. **De acordo com as categorías do censo do IBGE para raça ou cor, você se declara:**

( ) Indigena

( ) Amarelo (a)

( ) Preto (a)

( ) Pardo (a)

( ) Branco(a)

( ) Prefiro não responder

1. **Faixa etária**

( ) 21 - 30 anos

( ) 31 - 40 anos

( ) 41 - 50 anos

( ) 51 - 60 anos

( ) Acima de 60 anos

**6. Você desenvolveu algum sinal e/ou sintoma do COVID-19?**

( ) Tive sintomas

( ) Não tive sintomas

( ) Não sei se tive sintomas

**7. Você foi diagnosticado com o vírus (SARS-CoV-2)?**

( ) Sim, fiz o teste e o diagnóstico foi positivo

( ) Não, fiz o teste e o diagnóstico foi negativo

( ) Tive sintomas mas não consegui fazer o teste

**8. Alguém da sua família ou equipe de trabalho desenvolveu algum sintoma ou foi diagnosticado com COVID-19?**

( ) Sim

( ) Não

( ) Não sabe

**DIMENSÃO 2 – CARACTERÍSTICAS EDUCACIONAIS DA POPULAÇÃO ENTREVISTADA**

**1. Área de atuação:** (Você pode escolher mais de uma opção como resposta)

( ) Dentística Restauradora

( ) Odontologia Legal

( ) Pediatria

( ) Ortodontia e Ortopedia Facial

( ) Patologia Bucal

( ) Prótese Buco Maxilo Facial

( ) Prótese Dentária

( ) Saúde Coletiva e da Família

( ) Radiologia Odontológica e Imaginologia

( ) Disfunção Temporo-Mandibular

( ) Odontologia do Trabalho

( ) Odontologia para Pacientes com Necessidades Especiais

( ) Ortopedia Funcional dos Maxilares

( ) Gestão

( ) Odontologia Hospitalar

( ) Ensino

**2. Grau de formação concluído:**

( ) Clínico Geral

( ) Especialista

( ) Mestrado

( ) Doutorado

( ) Pós-doutorado

**3. Qual seu tempo de experiência no trabalho ou na especialidade q você exerce?**

( ) Até 4 anos

( ) 5 a 10 anos

( ) 11 a 20 anos

( ) 21 ou mais

( ) Não atuo clinicamente

**4. Onde você trabalha? (Você pode escolher mais de uma opção como resposta)**

( ) Hospital Publico

(...) Hospital privado

(...) UBS/AMA

(...) Clínica particular

(...) Policlínica

(...) Laboratório de Pesquisa

(...) Clínica Escola/ Faculdade

(...) Outro

**5. Conhece os protocolos de atendimento e biossegurança relacionado ao COVID-19?**

( ) Sim, todos

( ) Alguns

( ) Apenas dentro da minha especialidade

( ) Quase nada

( ) Nenhum

**6. Você recebeu algum treinamento para o uso, manutenção e descarte de EPIs? Se sim, onde foi esse treinamento? Mais de uma opção poderá ser marcada. Se você marcou "não" na pergunta acima, marque "não se aplica".**

( ) Sim

( ) Não

( ) Graduação

( ) Pós-graduação

( ) Na instituição/local em que trabalha

( ) Em outra instituição que já trabalhou

( ) Por conta própria

( ) Vi um vídeo de treinamento

( ) Sigo os protocolos de um Ebook

( ) Não se aplica

**7. Você consulta os sites dos conselhos de classe (CRO, CFO, entre outros) para busca de informações, protocolos clínicos e de biossegurança relacionados ao COVID – 19 ?**

( ) Sim

( ) Não

**8. Onde você costuma buscar informações sobre Biossegurança e Descarte de Resíduos de Saúde?**

( ) Livros

( ) Periódicos

( ) Protocolos dos Conselhos de Classe

( ) Internet

( ) Outros

**DIMENSÃO 3 – CARACTERÍSTICAS CLÍNICAS / DE TRABALHO E VARIÁVEIS ECONÔMICAS**

**1. Se sente apto a atender seus pacientes com estas informações?**

( ) Sim, totalmente

( ) Sim, mas com dúvidas

( ) Não

**2. Qual alternativa descreve o que você sente quanto ao seu trabalho; mais de uma opção pode ser marcada como resposta:**

( ) Preciso voltar ao trabalho para poder pagar minhas contas

( ) Preciso voltar ao trabalho por que eu amo minha profissão e não consigo ficar sem trabalhar, em casa

( ) Só vou voltar ao trabalho se meu patrão exigir ou cortar meu salário

( ) Tenho medo de pegar a doença atendendo meus pacientes

( ) Não tenho medo de pegar a doença atendendo meus pacientes

( ) Não parei de trabalhar durante a Pandemia

**3. Na sua rotina de atendimento, com relação ao uso de EPIs, assinale o que é verdadeiro:**

**RESPOSTAS EM BOLINHAS**

**Não uso**

**Já utilizava e vou continuar a utilizar**

**Passei a utilizar após a pandemia**

**Tenho utilizado por falta de outra opção no mercado**

Máscara cirúrgica, com tripla em TNT

Máscara N95

Máscara de tecido

Gorro descartável

Gorro de tecido

Óculos de proteção

Luvas de procedimento (descartáveis)

Avental de tecido

Avental descartável

Máscara facial acrílica/ FACE SHIELD

Pro pé

Luva Cirúrgica

Outros___________________________

**4. O que você tem feito, ou considera importante fazer no ambiente de atendimento odontológico, como medida de prevenção na transmissão do coronavírus (Você pode escolher mais de uma opção como resposta)**

( ) Limpeza frequente das mãos, utilizando álcool em gel ou água e sabão

( ) Rotina de limpeza e desinfecção de superfícies que foram expostas à pacientes suspeitos ou não

( ) Espaçamento entre os atendimentos agendados, evitando assim que os pacientes não se encontrem na sala de espera

( ) Orientação do uso de máscara em pacientes e acompanhante (quando indispensável)

( ) Uso de EPI por todos os integrantes da equipe

( ) Isolar paciente com a doença ou com suspeita em salas individuais adequadamente ventiladas

(...) Limpeza terminal diária

**5. Considerando os casos que tem se apresentado como demanda espontânea ou urgência odontológica durante a pandemia de COVID-19, você:**

( ) Considera atender clinicamente todos os casos porque se o paciente julgar necessário, sua vontade está em primeiro lugar

( ) Considera atender clinicamente todos os casos por motivos financeiros

( ) Considero atender os casos de urgência em compromisso ético ao bem estar do paciente

( ) Não considera atender qualquer caso porque o risco de contágio é muito grande

( ) A teleodontologia irá ser vista como uma melhor alternativa, se difundindo rapidamente e em maior escala

**6. Com relação ao seu trabalho, devido a chegada da pandemia COVID-19, qual dessas opções MAIS SE APROXIMA da sua situação ATUAL?**

( ) Permaneci atendendo e não houve mudança na minha rotina

( ) Adaptei meus serviços e não houve mudança na minha vida

( ) Não estou mais atendendo

( ) Estou em férias

( ) Estou trabalhando em home office

( ) Tive a jornada de trabalho e o salário reduzidos em 50%

( ) Tive a suspensão do contrato de trabalho com pagamento de seguro-desemprego

( ) Continuo trabalhando (nada foi alterado)

( ) Pedi demissão

( ) Fui demitido

( ) Outro

**7. A empresa e/ou local onde você trabalha:**

( ) Está aberta normalmente para o público

( ) Está aberta parcialmente para o público

( ) Está fechada para o público

( ) Está interditada/com acesso restrito de circulação de pessoas devido as medidas de distanciamento social/quarentena

( ) Não se aplica

**8. Durante a pandemia as condições de trabalho e renda são:**

( ) Melhores que a anterior

( ) Iguais a anterior

( ) Piores que a anterior

**9. Em relação à sua renda:**

( ) Não houve redução

( ) Houve redução de até 10%

( ) Houve redução acima de 10% até 50%

( ) Houve redução acima de 50% até 100%

**10. As previsões econômicas apontam para um período de recessão. Diante disso, com base em suas reservas econômicas atuais, por quanto tempo você conseguirá se manter?**

( ) Não tenho reserva econômica

( ) Menos de 1 mês

( ) 1 mês

( ) 2 meses

( ) 3 meses

( ) 4 meses

( ) 5 meses

( ) 6 meses

( ) Mais de 6 meses
