# Supplementary material for: COVID-19 pandemic impact on dentists in Latin America’s epicenter: São-Paulo, Brazil
Source: PLoS One. 2021 Aug 26;16(8):e0256092. doi: 10.1371/journal.pone.0256092 (PMC8389449; doi:10.1371/journal.pone.0256092)
Supplement: S3 Table — (English). (DOC) [file pone.0256092.s003.doc]

**S3 Table. Translated Questionnaire (English)**

**DIMENSION 1 – SOCIODEMOGRAPHIC CHARACTERISTICS OF THE INTERVIEWED POPULATION**

**Email: (Optional)**

**1. Which city do you live?**

**2. Which city do you work?**

**3. How do you identify yourself?**

( ) Man

( ) Woman

( ) Other

**4. According to the IBGE census categories for race or color, you declare yourself**

( ) Indigenous

( ) Yellow

( ) Black

( ) Brown

( ) White

( ) Rather not answer

**5. Age group**

( ) 21 - 30 years old

( ) 31 - 40 years old

( ) 41 - 50 years old

( ) 51 - 60 years old

( ) Over 60 years old

**6. Have you developed any signs and / or symptoms of COVID-19?**

( ) I had symptoms

( ) I had no symptoms

( ) I don't know if I had symptoms

**7. Were you diagnosed with the virus (SARS-CoV-2)?**

( ) Yes, I got tested and the diagnosis was positive

( ) No, I got tested and the diagnosis was negative

( ) I had symptoms but I couldn't get tested

**8. Has anyone in your family or work team developed any symptoms or been diagnosed with COVID-19?**

( ) Yes

( ) Not

( ) I don’t know

**DIMENSION 2- - EDUCATION CHARACTERISTICS OF THE INTERVIEWED POPULATION**

**1. Careers in Dentistry (You can choose more than one option as an answer)**

( ) Operative Dentistry

( ) Dental Law and Ethics

( ) Pediatric Dentistry

( ) Orthodontics

( ) Oral Patology

( ) Oral Maxillofacial Prosthesis

( ) Dental prosthesis

( ) Family Health

( ) Dental Radiology and Imaging

( ) Temporo-Mandibular Dysfunction

( ) Occupational Dentistry

( ) Dentistry for Patients with Special Needs

( ) Functional Jaw Orthopedics

( ) Management

( ) Hospital Dentistry

( ) Teaching

**2. Education**

( ) General Dentistry

( ) Certificate

( ) Master’s degre

( ) PhD

( ) Pos-doctoral training

**3. For how long have you worked in clinical pratice?**

( ) Up to 4 years

( ) 5 to 10 years

( ) 11 to 20 years

( ) 21 or more

( ) I am not in clinical practice

**4. Where do you work? (You can choose more than one option as an answer)**

( ) Public Hospital

(...) Private Hospital

(...) Basic Health Unit

(...) Private Dental Clinic

(...) Policlínica

(...) Research Laboratory

(...) Dental School

(...) Other

**5. Do you know the dental practice and biosafety protocols related to COVID-19?**

( ) Yes, all

( ) Some

( ) Only within my specialty

( ) Almost nothing

( ) None

**6. Did you receive any training on the use, maintenance and disposal of PPE? If so, where was this training? More than one option can be checked. If you checked "no" in the question above, check "does not apply".**

( ) Yes

( ) Not

( ) Graduate school

( ) Postgraduate studies

( ) In the institution / place where I work

( ) In another institution that I have worked

( ) On my own

( ) I saw a training video

( ) I follow the protocols of an Ebook

( ) Does not apply

**7. Do you consult the dental council websites (CRO, CFO, among others) for information, clinical and biosafety protocols related to COVID - 19?**

( ) Yes

( ) No

**8. Where do you usually look for information on Biosafety and Health Waste Disposal?**

( ) Books

( ) Journals

( ) Dental Council Protocols

( ) Internet

( ) Others

**DIMENSION 3 - CLINICAL/WORK CHARACTERISTICS AND ECONOMICAL VARIABLES**

**1.Do you feel able to assist your patients with this information?**

( ) Yes, totally

( ) Yes, but with doubts

( ) No

**2. Which alternative describes how you feel about your job; more than one option can be marked as an answer:**

( ) I need to go back to work to be able to pay my bills

( ) I need to go back to work because I love my profession and I can't stay without work, at home

( ) I will only go back to work if my boss demands or cuts my salary

( ) I am afraid of getting the disease by seeing my patients

( ) I am not afraid of catching the disease seeing my patients

( ) I didn't stop working during the Pandemic

**3. In your dental practice routine, in relation to the use of PPE, mark what is true:**

**DOTTED ANSWERS**

I do not use

I used it and I will continue to use it

I started using after the pandemic

I have used it for lack of another option on the market

Surgical mask with triple TNT

Mask N95

Fabric mask

Disposable hat

Fabric hat

Protective goggles

Procedural gloves (disposable)

Fabric lab coat

Disposable lab coat

Acrylic face mask / FACE SHIELD

Disposable foot cover

Surgical Glove

Others___________________________

**4.What have you done, or do you consider important to do in the dental practice environment, as a preventive measure in the transmission of coronavirus (You can choose more than one option as an answer)**

( ) Frequent hand cleaning using hand sanitizer or soap and water

( ) Routine cleaning and disinfection of surfaces that have been exposed to patients

( ) Spacing between scheduled appointments, thus preventing patients from being in the waiting room

( ) Guidance on the use of a mask to patients and companions (when necessary)

( ) Use of PPE by all team members

( ) Isolate patient with the disease or with suspicion in individual rooms adequately ventilated

( ) Daily end of the day cleaning

**5. Considering the cases that have been presented as spontaneous demand or dental urgency during the COVID-19 pandemic, you:**

( ) Considers providing clinical care to all cases because if the patient deems it necessary, his will comes first

( ) Considers providing clinical care to all cases for financial reasons

( ) I consider providing clinical care to urgent cases in an ethical commitment to the patient's well-being

( ) Does not consider providing clinical care any case because the risk of contagion is very high

**6. Regarding your work, due to the arrival of the COVID-19 pandemic, which of these options IS MOST APPROXIMATE to your CURRENT situation?**

( ) I continued providing dental care and there was no change in my routine

( ) I adapted my services and there was no change in my life

( ) I'm not providing dental care anymore

( ) I'm on vacation

( ) I am working from home

( ) I had my workday and salary reduced by 50%

( ) I had the employment contract suspended with payment of unemployment insurance

( ) I keep working (nothing has changed)

( ) I quit

( ) I was fired

( ) Other

**7. The company and / or place where you work:**

( ) It is normally open to the public

( ) It is partially open to the public

( ) It is closed to the public

( ) It is forbidden / with restricted access for the circulation of people due to the measures of social distance / quarantine

( ) Not applicable

**8. During the pandemic, working and income conditions are:**

( ) Better than the previous one

( ) Same as previous

( ) Worse than the previous

**9. Regarding your income:**

( ) There was no reduction

( ) There was a reduction of up to 10%

( ) There was a reduction above 10% up to 50%

( ) There was a reduction above 50% up to 100%

**10. Economic forecasts point to a period of recession. Given that, based on your current savings, how long will you be able to financily maintain yourself?**

( ) I have no savings

( ) Less than 1 month

( ) 1 month

( ) 2 months

( ) 3 months

( ) Four months

( ) 5 months

( ) 6 months

( ) More than 6 months
