# Supplementary material for: COVID-19 pandemic impact on dentists in Latin America’s epicenter: São-Paulo, Brazil
Source: PLoS One. 2021 Aug 26;16(8):e0256092. doi: 10.1371/journal.pone.0256092 (PMC8389449; doi:10.1371/journal.pone.0256092)
Supplement: Attachment — Submitted filename: PONE-D-20-40253_reviewed 12 March.pdf [file pone.0256092.s005.pdf]

PLOS ONE

COVID-19 pandemic impact on dentists in Latin America's epicenter: São-Paulo, Brazil

--Manuscript Draft--

**Manuscript Number:**	PONE-D-20-40253
**Article Type:**	Research Article
**Full Title:**	COVID-19 pandemic impact on dentists in Latin America's epicenter: São-Paulo, Brazil
**Short Title:**	COVID-19 pandemic impact on dentists
**Corresponding Author:**	Debora Heller, PhD<br>Universidade Cruzeiro do Sul<br>Sao Paulo, BRAZIL
**Keywords:**	Coronavirus; SARS-CoV-2; COVID-19; Dentistry
**Abstract:**	The state of São Paulo, Brazil, where more than 94.000 dentists are currently registered, has become the epicenter of COVID-19 in Latin America. The aim of this cross-sectional study was to evaluate the impact of COVID-19 pandemic on dentists in this state. A semi-structured questionnaire was sent via e-mail to all dentists registered in the Dental Council of São Paulo (CROSP). The impact of COVID-19 pandemic was assessed through questions related to demographic, socioeconomic, dental practice characteristics and personal protective equipment (PPE) use. Ordinal logistic regression analysis was performed to investigate the association between all the variables (p<0.05). Over 8 days, 2113 responses were received. Only 26.52% of the sample reported a low-income reduction (from 0-10%), while the majority of dentists reported a more negative financial impact, 35.6% with a reduction of more than 50% of their monthly income. Dentists who worked in the private sector and at the capital had a greater financial impact when compared to those of the public sector and countryside of the state (p<0.05). Furthermore, the use of PPE by dentists has grown during the pandemic. This study provides evidence of the negative impact of the COVID-19 pandemic on dentistry in Brazil.
**Order of Authors:**	Tatiane Fernandes Novaes
	Maisa Camillo Jordão
	Carlos Felipe Bonacina
	André Oswaldo Veronezi
	Carlos Ariel Rodrigues de Araujo
	Isabel Cristina Olegário
	Daniele Boina de Oliveira
	Veranika Ushakova
	Alexander Birbrair
	Danielle da Costa Palacio
	Debora Heller, PhD
**Additional Information:**	
**Question**	**Response**
**Financial Disclosure**<br><br>Enter a financial disclosure statement that describes the sources of funding for the work included in this submission. Review the submission guidelines for detailed requirements. View published research	This research was funded by Instituto Serrapilheira, grant number Serra-1708-316 15285 (AB)-https://serrapilheira.org/en/; Pró-reitoria de Pesquisa/Universidade Federal de Minas Gerais, grant number 05/2016 (AB)-https://www.ufmg.br/prpq/; FAPESP, grant number 2013/07467-1 and 2017/17943-6 (AB)-https://fapesp.br/.<br>The funders had no role in study design, data collection and analysis, decision to publish, or preparation of the manuscript.

Powered by Editorial Manager® and ProduXion Manager® from Aries Systems Corporation

articles from *PLOS ONE* for specific examples.<br><br>This statement is required for submission and **will appear in the published article** if the submission is accepted. Please make sure it is accurate.<br><br>**Unfunded studies**<br>Enter: *The author(s) received no specific funding for this work.*<br><br>**Funded studies**<br>Enter a statement with the following details:<br>• Initials of the authors who received each award<br>• Grant numbers awarded to each author<br>• The full name of each funder<br>• URL of each funder website<br>• Did the sponsors or funders play any role in the study design, data collection and analysis, decision to publish, or preparation of the manuscript?<br>• **NO** - Include this sentence at the end of your statement: *The funders had no role in study design, data collection and analysis, decision to publish, or preparation of the manuscript.*<br>• **YES** - Specify the role(s) played.<br><br>* typeset	
**Competing Interests**<br><br>Use the instructions below to enter a competing interest statement for this submission. On behalf of all authors, disclose any competing interests that could be perceived to bias this work—acknowledging all financial support and any other relevant financial or non-financial competing interests.<br><br>This statement **will appear in the published article** if the submission is accepted. Please make sure it is accurate. View published research articles from *PLOS ONE* for specific examples.	The authors have declared that no competing interests exist.

**NO authors have competing interests**<br><br>Enter: *The authors have declared that no competing interests exist.*<br><br>**Authors with competing interests**<br><br>Enter competing interest details beginning with this statement:<br><br>*I have read the journal's policy and the authors of this manuscript have the following competing interests: [insert competing interests here]*<br><br>* typeset	
**Ethics Statement**<br><br>Enter an ethics statement for this submission. This statement is required if the study involved:<br><br>• Human participants<br>• Human specimens or tissue<br>• Vertebrate animals or cephalopods<br>• Vertebrate embryos or tissues<br>• Field research<br><br>Write "N/A" if the submission does not require an ethics statement.<br><br>General guidance is provided below. Consult the submission guidelines for detailed instructions. **Make sure that all information entered here is included in the Methods section of the manuscript.**	This work was conducted according to the Helsinki declaration. The project was previously approved by the ethics committee of the Universidade Cruzeiro do Sul under CAAE number: 31720720.9.0000.8084. In addition, all methods were performed in accordance with the relevant guidelines and regulations and the original datasets generated and/or analyzed during the current study are available from the corresponding author on reasonable request. The consent form was made available prior to the questionnaire, in writing and consent was given through an alternative in the online questionnaire. Only dentists who read the informed consent form and agreed to participate, were included in the study.

Powered by Editorial Manager® and ProduXion Manager® from Aries Systems Corporation

**Format for specific study types**<br><br>**Human Subject Research (involving human participants and/or tissue)**<br>• Give the name of the institutional review board or ethics committee that approved the study<br>• Include the approval number and/or a statement indicating approval of this research<br>• Indicate the form of consent obtained (written/oral) or the reason that consent was not obtained (e.g. the data were analyzed anonymously)<br><br>**Animal Research (involving vertebrate animals, embryos or tissues)**<br>• Provide the name of the Institutional Animal Care and Use Committee (IACUC) or other relevant ethics board that reviewed the study protocol, and indicate whether they approved this research or granted a formal waiver of ethical approval<br>• Include an approval number if one was obtained<br>• If the study involved *non-human primates*, add *additional details* about animal welfare and steps taken to ameliorate suffering<br>• If anesthesia, euthanasia, or any kind of animal sacrifice is part of the study, include briefly which substances and/or methods were applied<br><br>**Field Research**<br><br>Include the following details if this study involves the collection of plant, animal, or other materials from a natural setting:<br>• Field permit number<br>• Name of the institution or relevant body that granted permission	
**Data Availability**<br><br>Authors are required to make all data underlying the findings described fully available, without restriction, and from the time of publication. PLOS allows rare exceptions to address legal and ethical concerns. See the PLOS Data Policy and FAQ for detailed information.	Yes - all data are fully available without restriction

A Data Availability Statement describing where the data can be found is required at submission. Your answers to this question constitute the Data Availability Statement and **will be published in the article**, if accepted.<br><br>**Important:** Stating 'data available on request from the author' is not sufficient. If your data are only available upon request, select 'No' for the first question and explain your exceptional situation in the text box.<br><br>Do the authors confirm that all data underlying the findings described in their manuscript are fully available without restriction?	
**Describe where the data may be found in full sentences. If you are copying our sample text, replace any instances of XXX with the appropriate details.**<br><br>• If the data are **held or will be held in a public repository**, include URLs, accession numbers or DOIs. If this information will only be available after acceptance, indicate this by ticking the box below. For example: *All XXX files are available from the XXX database (accession number(s) XXX, XXX.).*<br>• If the data are all contained **within the manuscript and/or Supporting Information files**, enter the following: *All relevant data are within the manuscript and its Supporting Information files.*<br>• If neither of these applies but you are able to provide **details of access elsewhere**, with or without limitations, please do so. For example:<br><br>*Data cannot be shared publicly because of [XXX]. Data are available from the XXX Institutional Data Access / Ethics Committee (contact via XXX) for researchers who meet the criteria for access to confidential data.*<br><br>*The data underlying the results presented in the study are available from (include the name of the third party*	All relevant data are within the manuscript and its Supporting Information files.

and contact information or URL).<br>• This text is appropriate if the data are owned by a third party and authors do not have permission to share the data.<br><br>* typeset	
Additional data availability information:	

Manuscript Click here to access/download;Manuscript;articleCOVIDPLOSONE.docx

COVID-19 pandemic impact on dentists in Latin America's epicenter: São-Paulo, Brazil

Tatiane Fernandes Novaes[1], Maisa Camillo Jordão[1], Carlos Felipe Bonacina[1], André Oswaldo Veronezi[1], Carlos Ariel Rodrigues de Araujo[1], Isabel Cristina Olegário [2], Daniele Boina de Oliveira[3], Veranika Ushakova[4], Alexander Birbrair[4], Danielle da Costa Palacio[3,5], Debora Heller[1,3,6*]

1 Postgraduate Program in Dentistry, Cruzeiro do Sul University, São Paulo, Brazil.

2 Assistant Professor in Paediatric Dentistry, Dublin Dental University Hospital, Trinity College Dublin, Ireland.

3 Hospital Israelita Albert Einstein, São Paulo, Brazil.

4 Department of Pathology, Federal University of Minas Gerais, Belo Horizonte, Minas Gerais, Brazil.

5 School of Dentistry, Universidade Estadual de Campinas, Departamento de Odontologia Social, Piracicaba, Brazil.

6 Department of Periodontology, School of Dentistry, The University of Texas Health Science Center at San Antonio, San Antonio, EUA.

*Corresponding Author:

Débora Heller

Universidade Cruzeiro do Sul

Rua Galvão Bueno, 868,

São Paulo, São Paulo

Debora.heller@cruzeirodosul.edu.br

Abstract

The state of São Paulo, Brazil, where more than 94.000 dentists are currently registered, has become the epicenter of COVID-19 in Latin America. The aim of this cross-sectional study was to evaluate the impact of COVID-19 pandemic on dentists in this state. A semi-structured questionnaire was sent via e-mail to all dentists registered in the Dental Council of São Paulo (CROSP). The impact of COVID-19 pandemic was assessed through questions related to demographic, socioeconomic, dental practice characteristics and personal protective equipment (PPE) use. Ordinal logistic regression analysis was performed to investigate the association between all the variables ($p<0.05$). Over 8 days, 2113 responses were received. Only 26.52% of the sample reported a low-income reduction (from 0-10%), while the majority of dentists reported a more negative financial impact, 35.6% with a reduction of more than 50% of their monthly income. Dentists who worked in the private sector and at the capital had a greater financial impact when compared to those of the public sector and countryside of the state ($p<0.05$). Furthermore, the use of PPE by dentists has grown during the pandemic. This study provides evidence of the negative impact of the COVID-19 pandemic on dentistry in Brazil.

Keywords: Coronavirus; SARS-CoV-2; COVID-19; Dentistry;

Introduction

In early December 2019, an outbreak of pneumonia with an unknown cause emerged in Wuhan, Hubei (China). The clinical symptoms were similar to a viral pneumonia, including fever, dizziness, and cough (1). After sequencing analysis of samples obtained from the respiratory tract, the pathogen was identified as a new coronavirus (coronavirus 2, SARS-CoV-2, COVID-19), causing a severe acute respiratory syndrome due to phylogenetic similarity with SARS-CoV (2). COVID-19 infected cases have since grown exponentially, and as a result, the World Health Organization (WHO) declared a public health emergency of international interest (3). There are more than 68 million cases confirmed worldwide to date, with more than 1,5 million deaths (4). SARS-CoV-2 was first reported in Brazil on February 25th, 2020, and due to its exponential spread, the country has become the epicenter for COVID-19 in Latin America, with more than 6,6 million cases and more than 177 thousand deaths (4).

Considering that the virus is transmitted predominantly through aerosol and salivary droplets (5) and that dental interventions are performed close to the oropharyngeal region, dentists are exposed to a high risk of infection (6). In fact, due to this risk of widespread transmission of SARS-CoV-2 in dental practice, a suspension of elective care was imposed by national and international dental health entities (7) and new management protocols for dental practitioners have

been released (8). As a consequence, dentists worldwide reported seeing fewer patients, and an increase in the expenses on personal protective equipment (PPE). The higher demand for PPE has caused supplies shortages, including N95 face masks, that have become mandatory when dealing with aerosol procedures (9,10), contributing to the vulnerability of all dental care professionals.

The state of São Paulo, Brazil is considered the financial heart of Brazil and has become the epicenter of COVID-19 in Latin America (11). Currently, there are more than 94.000 dentists registered in the local Dental Council (12). Thus, our aim was to evaluate the impact of COVID-19 pandemic on dentists in São-Paulo, Brazil.

Methods

1.1.Ethical aspects

This work was conducted according to the Helsinki declaration. The project was previously approved by the ethics committee of the Universidade Cruzeiro do Sul under CAAE number: 31720720.9.0000.8084. In addition, all methods were performed in accordance with the relevant guidelines and regulations and the original datasets generated and/or analyzed during the current study are available from the corresponding author on reasonable request. The consent

form was made available prior to the questionnaire, in writing and consent was given through an alternative in the online questionnaire. Only dentists who read the informed consent form and agreed to participate, were included in the study.

1.2.Study design and data collection instrument

The STROBE checklist was (13) followed to show transparency in the analysis and reporting of this observational study (Supplemental Table 1). In addition, SURGE guideline (The SUrvey Reporting GuidelinE) was used for reporting the methods and results of the present survey (14).

A cross-sectional study was designed and a semi-structured questionnaire was sent to all dentists registered in the Regional Dental Council of São Paulo (CROSP), Brazil, who have email addresses cataloged in their database. In addition, an Instagram disclosure and WhatsApp messages were also sent in order to enhance dentist's participation and encourage them to check their e-mail inbox.

In order to certify a good reliability and validity of the questionnaire and its dimensions, we conducted a pre-test in a sample of 10 dentists. The pre-testers were asked to answer the questionnaire, and give their feedback on its clarity and record the time spent to complete all questions. The authors discussed all questions until reaching a consensus. Pre-testers were not eligible to participate

in this study, since they were dentists from public and private sectors from others states in Brazil.

1.2. *Data collection instrument*

The questionnaire was hosted online (Google Forms) and consisted of 26 questions, divided into 3 main groups: Dimension 1 – Sociodemographic characteristics of the interviewed population (8 questions), including its inclusion or not in SARS-Cov-2 risk groups; Dimension 2 - Education characteristics of the interviewed population, including information/courses regarding safety protocols for clinical practice (8 questions); Dimension 3 – Clinical/Work characteristics and economical variables (10 questions) The original questionnaire and a translation of the questionnaire are presented in Supplemental Tables 2 and 3.

1.3. *Statistical analysis*

Data were analyzed using Stata/SE 16.11 statistical software (StataCorp LP, College Station, Texas, USA). Descriptive analysis was used to describe general, socioeconomic and dental practice characteristics of the interviewed population (n/%). An alluvial diagram was built using RAWGraphs software to compare the distribution between workplace (public/private/both) with financial impact. Unadjusted and adjusted ordinal logistic regression analysis were performed to

investigate the association between the financial impact (income reduction: 0-10%; 10-50%; >50% reduction) and independent variables. Forwards stepwise ordinal logistic regression was performed using a p-value <0.20 as the criteria for inclusion in the adjusted model, and only variables with a p-value <0.05 were kept in the final model. Estimates of odds ratio are shown with 95% confidence intervals.

Results

From total of 93.280 dentists invited to participate by e-mail, 2,348 accessed the Google forms link provided from June 25, 2020 until July 02, 2020, and from those, 2.113 accepted to participate after reading the informed consent form. Only 4.6% (n=101) opted to not participate in the present study.

Dentists from the countryside (43%) and from the capital (57%) answered the questionnaire. The majority of the respondents were female (n=1,565; 74%) and white (n=1,837; 87%). In terms of educational level, 29% were general dentists, 50% had a certificate degree and 21% had a graduate degree (MSc, PhD and/or Postdoc). Almost 70% of the respondents had more than 10 years of clinical experience and did not belong to any COVID-19 risk group. While the vast majority (98,91%) of subjects reported to have knowledge about COVID-19, only 16.8% received any specific training on the use, maintenance, and disposal of

personal protective equipment (PPE). The distribution of all sociodemographic, education and work characteristics of the sample is found in Table 1.

Table 1. Sociodemographic, educational, and work characteristics of the sample (n=2,113).

Variable/Category	n	%
**Sociodemographic**		
**Gender**		
Female	1,565	74.07
Male	547	25.89
Other	1	0.05
**IBGE´s# race classification**		
Indigenous	0	0
Yellow	126	5.96
Brown	133	6.29
Black	0	0
White	1,837	86.94
I prefer not to answer	17	0.80
**Age range**		
20-30 years old	398	18.84
31-40 years old	517	24.47
41-50 years old	684	32.37
51-60 years old	382	18.08
>60 years old	132	6.25
**Leaves in**		
São Paulo	911	43.11
Countryside	1,202	56.89
**COVID-19 Risk group**		
Yes	521	24.66
No	1,563	73.97

I do not know	29	1.37
**Education Characteristics**		
**Higher educational level**		
Dentistry degree	613	29.01
Specialization degree	1,067	50.50
Master's degree	274	12.97
Doctorate degree	109	5.16
Postdoctoral degree	50	2.37
**Time since graduation (clinical experience in years)**		
<4 years	326	15.43
5-10 years	297	14.06
11-20 years	558	26.41
>20 years	915	43.30
I do not work in clinical practice	17	0.80
**COVID -19 Education Characteristics**		
**Do you search for information in the Dental Council website?**		
Yes	1,647	77.95
No	453	21.44
Not answered	13	0.62
**Do you acknowledge COVID-19 health safety protocols for clinical practice?**		
Yes	1,264	59.82
Some	826	39.09
None, almost none.	23	1.09
**Have you received any training on the use, maintenance, and disposal of PPE*?**		
Yes	355	16.80
No	1,758	83.20
**Where?**		
Yes, during undergraduate or postgraduation course	275	13.01
Yes, in my workplace (past or present)	164	7.76
Yes, on my own or internet/ other medias	861	40.75
Yes, more than one of the above	368	17.42

No, I did not take any course	355	16.80
Yes, but did not specify where/how.	90	4.26
**Work characteristics**		
**Where do you work?**		
São Paulo (capital)	878	41.69
Countryside	1,164	55.27
Both	64	3.04
**Public/Private sector**		
Public	239	11.31
Private	1,589	75.20
Both	285	13.49
**Work routine**		
Remains the same/I have adapted to the new reality	1,447	68.51
I am on holidays/not on practice	221	10.46
Working from home	100	4.73
I got fired or resigned	35	1.66
Other	309	14.63
**Workplace operation**		
Open as usual	606	28.69
Partially open/Restricted access	1,171	55.45
Closed	154	7.29
Other	181	8.57
**Income Reduction**		
No income reduction/ less than 10%	560	26.52
Income reduction between 10-50%	801	37.93
More than 50% of income reduction	751	35.56
**Total**	**2,113**	**100**

*** PPE = Personal Protective Equipment; #IBGE: Brazilian Institute of Geography and Statistics**

Regarding their professional activities, 11.3% of respondents worked in public sector, while 75.2% were in private sector and 13.5% worked in both. Although most practices and workplaces had already adapted to the new national guidelines (n=1,447; 68.5%), most of them were only partially open or working with restricted access (n=1,171; 55.5%) (Table 1).

COVID-19 pandemic had a detrimental financial impact on dental practice – only 26.5% (n=560) respondents reported no income reduction or a reduction lower than 10%, while 37.9% had a reduction between 10-50% and 35.6% had more than 50% of income reduction. Table 2 shows the results of ordinal logistic regression of the financial impact and associated variables. Variables such as gender, race, belonging to COVID-19 risk group, educational level and clinical experience did not influence income reduction ($p>0.05$). However, dentists aged between 31 and 40 years old experienced a steeper financial burden when compared to the younger age group (20-30 years old; OR=0.77; CI=0.60-0.98). Also, dentists who worked only in the countryside reported 16% less financial contraction compared to dentists working in the state's capital ($p=0.041$). Workplaces that were either partially open, had restricted access or were closed showed a negative influence on the reported economic impact when compared to workplaces that were opened as usual ($p<0.001$). Additionally, dentists from the private sector reported suffering 2.41 times greater financially than dentists

from the public sector (CI=1.84-3.14; p<0.001). Likewise, dentists who work in both public and private sectors had higher detrimental impact than those who worked only in the public sector (OR=1.51; p=0.013). Figure 1 shows the alluvial diagram showing the distribution of the financial impact among public and private sectors.

Table 2. Descriptive and Ordinal logistic regression analysis of the financial impact (percentage of income reduction) and independent variables.

**Variable/Category**	**Financial Impact**			**Ordinal logistic regression analysis**			
	**0-10%**	**10-50%**	**>50%**	**Unadjusted**		**Adjusted**	
	**n (%)**	**n (%)**	**n (%)**	**OR (CI 95%)**	**p-value**	**OR (CI 95%)**	**p-value**
**Gender**							
Female (ref)	418 (26.73)	593 (37.92)	553 (35.36)	1			
Male	141 (25.78)	208 (38.03)	198 (36.20)	1.04 (0.87-1.24)	0.648	-	-
**IBGE´s# race classification**							
White (ref)	483 (26.31)	711 (38.73)	642 (34.97)	1			
Brown/Black	34 (25.56)	54 (40.60)	45 (33.83)	0.98 (0.71-1.36)	0.943	-	-
Yellow	37 (29.37)	33 (26.19)	59 (44.44)	1.20 (0.85-1.70)	0.298	-	-
I prefer not to answer	6 (35.29)	3 (17.65)	8 (47.06)	1.13 (0.43-2.93)	0.803	-	-
**Age range**							
20-30 years old	94 (23.62)	152 (38.19)	152 (38.19)	1		1	
31-40 years old	148 (28.63)	210 (40.62)	159 (30.75)	0.74 (0.59-0.94)	0.017*	0.77 (0.60-0.98)	0.040*
41-50 years old	184 (26.94)	260 (38.07)	239 (34.99)	0.85 (0.68-1.07)	0.190	0.89 (0.71-1.12)	0.405

>50 years old	134 (26.07)	179 (34.82)	201 (39.11)	0.97 (0.76-1.23)	0.819	1.00 (0.78-1.49)	0.758
**COVID-19 Risk group**							
Yes	140 (26.87)	185 (35.51)	196 (37.62)	1			
No	414 (26.50)	603 (38.60)	545 (34.89)	0.94 (0.78-1.13)	0.517	-	-
I do not know	6 (20.69)	13 (44.83)	10 (34.48)	1.05 (0.53-2.06)	0.881	-	-
**Education Characteristics**							
**Higher educational level**							
Dentistry degree	158 (25.77)	220 (35.89)	235 (38.34)	1			
Certificate degree	280 (26.24)	407 (38.14)	380 (35.61)	0.92 (0.76-1.11)	0.397	-	-
MSc/PhD/Postdoc	122 (28.24)	174 (40.28)	136 (31.48)	0.79 (0.63-1.00)	0.051	-	-
**Time since graduation (clinical experience in years)**							
<4 years/not working in clinical practice	84 (24.49)	120 (34.99)	139 (40.52)	1			
5-10 years	71 (23.91)	130 (43.77)	96 (32.32)	0.82 (0.61-1.09)	0.180	-	-
11-20 years	155 (27.83)	219 (39.32)	183 (32.85)	0.76 (0.59-0.98)	0.036*	-	-
>20 years	250 (27.32)	332 (36.28)	333 (36.39)	0.84 (0.67-1.06)	0.153	-	-
**Work characteristics**							
**Where do you work?**							
São Paulo (capital)	218 (24.83)	322 (36.67)	338 (38.50)	1		1	
Countryside	322 (27.69)	454 (39.04)	387 (33.28)	0.82 (0.70-0.96)	0.019*	0.84 (0.72-0.99)	0.041*
Both	18 (28.13)	23 (35.94)	23 (35.94)	0.87 (0.54-1.39)	0.567	0.92 (0.56-1.49)	0.743
**Public/Private sector**							
Public	108 (45.19)	72 (30.13)	59 (24.69)	1			
Private	359 (22.61)	619 (38.98)	610 (38.41)	2.43 (1.87-3.15)	<0.001*	2.41 (1.84-3.14)	<0.001*
Both	93 (32.63)	110 (38.60)	82 (28.77)	1.53 (1.10-2.11)	0.010*	1.51 (1.09-2.11)	0.013*
**Workplace operation**							

Open as usual	226 (37.29)	233 (38.45)	147 (24.26)	1		1	
Partially open/ Restricted access	259 (22.12)	483 (41.25)	429 (36.64)	1.92 (1.59-2.29)	<0.001*	1.90 (1.58-2.28)	<0.001*
Closed	45 (29.22)	41 (26.62)	68 (44.16)	2.01 (1.43-2.83)	<0.001*	1.92 (1.35-2.71)	<0.001*
Other	30 (16.57)	44 (24.31)	107 (59.12)	4.18 (3.00-5.82)	<0.001*	4.15 (2.98-5.79)	<0.001*
**Total**	560 (26.52)	801 (37.93)	751 (35.56)				

IBGE: Brazilian Institute of Geography and Statistics; MSc: Master of Sciences; PhD: Doctor of philosophy; OR= Odds Ratio; CI 95% = Confidence Interval 95%; *p<0.05

Figure 1. Alluvial diagram of workplace (public, private or both) and financial impact (income reduction 0-10%; 10-50% and >50%).

Regarding the use of PPE for infection control, the majority of dentists reported using N95 masks, face shields, disposable gowns and shoe covers (Figure 2).

Figure 2. Distribution of the Personal Protective Equipment (PPE) for Infection Control used.

Discussion

The present study investigated the impact of COVID-19 pandemic on dentists practicing in the state of São Paulo, Brazil, which has more than 639.000 confirmed cases with more than 25.000 deaths (15). We reported a detrimental

financial impact of COVID-19 pandemic on dentists, and provided data on the sociodemographic, educational profile, dental practice characteristics and personal protective equipment (PPE) use by dentists during this time.

The majority (73.50%) of dentists reported a reduction of up to half the income (37.90%) or more than half of the income (35.60%), with this being more prevalent among dentists working in the private sector. This result is in accordance with other studies (16–23), that although with a smaller sample size, have also detected a negative financial impact of COVID-19 pandemic in dentistry worldwide. Although the present study focused on dentists, it is important to aknowledge the possible impact of the pandemic on all other professionals working in the dental office, such as dental hygienists, dental assistants. A recent study reported that only 27% of american dental surgeons were able to offer full payment to their employees (22).

Dentists are among the top at-risk occupations for contracting COVID-19 (6). Concerning the use of PPE for infection control, our data showed that the majority of the respondents adopt it. It is possible that these add additional costs to the dental practices, justifying a higher financial burden on the private sector when the dentists spend their own resources to purchase these items. Furthermore, dentists also reported difficulties finding PPEs and some resorted

to using reusable masks, in agreement with known bottlenecks for accessibility to PPE, that includes supply chain shortages (24,25).

Unfortunately, 83.2% of the participants in this study reported not having received any specific training to control COVID-19 transmission in the healthcare environment. Although there are plenty of free courses and several ways of continuing education which were widely publicized, such as courses from the Open University in the Brazilian Unified Health System (26) and other free access resources in philanthropic institutions (27), most respondents did not access this information. On the other hand, among dentists that reported having received training on the use, maintenance, and disposal of PPE, 40.75% did so through the internet or other social media. The rate of dissemination of traditional peer reviewed publications, static websites and emails are known to be slower and generally in English language (28).

Dentists of the private network have their remuneration determined by the attendance and procedures performed, either directly, through private offices and clinics, or indirectly, through the provision of services for agreements and cooperatives. On the other hand, dental professionals from the brazilian public network generally present labour contracts or statutory regime, with fixed remuneration and benefits. Consequently, even with the activities partially paralyzed and the attendance focused only on dental emergency cases, the public

sector reorganized its work processes to face the pandemic, expanding the field of action of dental professionals directing them to different functions such as: telemonitoring of confirmed cases of COVID, administrative work, epidemiological inquiries, research, among others (29). All these factors can explain why the private sector dentists included in the present investigation suffered twice the financial impact when compared to public sector dentists.

As the largest country in South America and the fifth largest nation in the world, the COVID-19 pandemic reached different regions in Brazil at different times and proportions (11). At the time this questionnaire was sent, there were fewer cases of COVID-19 in the countryside, which can explain why dentists from the countryside experienced less impact when compared to dentists from the capital.

It is important to recognize some limitations of the present study, such as the convenience sample, which included dentists who had access to their emails and social media in the period that the questionnaire was released. Further larger population studies are needed in different countries to understand the COVID-19 impact on dentistry.

5. Conclusions

We presented evidence of the negative impact of COVID-19 pandemic on dentists in the State of São Paulo, Brazil. Brazilian dentists reported an income reduced by more than 50% and the dental private sector shouldering twice the burden of those in the public sector. While these are unprecedented challenging times, we hope this study will help dental and other health care professionals to better understand the consequences of disease in dental settings and strengthen preparedness throughout the dental health care system.

Funding: This research was funded by Instituto Serrapilheira, grant number Serra-1708-316 15285; Pró-reitoria de Pesquisa/Universidade Federal de Minas Gerais, grant number 05/2016; FAPESP, grant number 2013/07467-1 and 2017/17943-6.

Acknowledgments: A.V., C.F.B. and C.A.R.A. received a scholarship from Coordination for the Improvement of Higher Education Personnel (CAPES).

CRediT author statement

Tatiane Fernandes Novaes: Conceptualization, Methodology, Validation, Formal analysis, Investigation, Data Curation, Writing - Original Draft, Visualization.

Maisa Camillo Jordão: Conceptualization, Methodology, Validation, Formal analysis, Investigation, Data Curation, Writing - Original Draft, Visualization.

Carlos Felipe Bonacina: Methodology, Investigation, Writing - Original Draft. **André Oswaldo Veronezi:** Methodology, Investigation, Writing - Original Draft. **Carlos Ariel Rodrigues de Araujo:** Writing - Review & Editing. **Isabel Cristina Olegário:** Formal analysis, Writing - Original Draft**. Daniele Boina de Oliveira:** Formal analysis, Writing - Original Draft**. Veranika Ushakova:** Formal analysis, Writing - Review & Editing**. Alexander Birbrair:** Formal analysis, Writing - Review & Editing**. Danielle da Costa Palacio:** Conceptualization, Methodology, Validation, Formal analysis, Investigation, Data Curation,Writing - Original Draft, Visualization. **Debora Heller:** Conceptualization, Methodology, Validation, Formal analysis, Investigation, Data Curation, Writing - Review & Editing, Visualization, Supervision.

Conflicts of Interest: Authors declare no conflict of interest.

References

1. Huang H, Zhang M, Chen C, Zhang H, Wei Y, Tian J, et al. Clinical characteristics of COVID-19 in patients with preexisting ILD: A retrospective study in a single center in Wuhan, China. J Med Virol. 2020;
2. Jin Y, Wang M, Zuo Z, Fan C, Ye F, Cai Z, et al. Diagnostic value and dynamic variance of serum antibody in coronavirus disease 2019. Int J Infect Dis. 2020;
3. World Health Organization. Coronavirus Disease (COVID-19)- Weekly Epidemiological Update at 05/10/2020. World Heal Organ Geneva, Switz. 2020;
4. Dong E, Du H, Gardner L. An interactive web-based dashboard to track COVID-19 in real time. The Lancet Infectious Diseases. 2020.
5. Izzetti R, Nisi M, Gabriele M, Graziani F. COVID-19 Transmission in Dental Practice: Brief Review of Preventive Measures in Italy. Journal of Dental Research. 2020.
6. Ather A, Patel B, Ruparel NB, Diogenes A, Hargreaves KM. Coronavirus Disease 19 (COVID-19): Implications for Clinical Dental Care. Journal of

Endodontics. 2020.

7. Centers for Disease Control and Prevention. Guidance for Dental Settings. Interim Infection Prevention and Control Guidance for Dental Settings During the COVID-19 Response. Centers for Disease Control and Prevention. 2020.

8. Meng L, Hua F, Bian Z. Coronavirus Disease 2019 (COVID-19): Emerging and Future Challenges for Dental and Oral Medicine. J Dent Res. 2020;

9. Amato A, Caggiano M, Amato M, Moccia G, Capunzo M, De Caro F. Infection control in dental practice during the covid-19 pandemic. Int J Environ Res Public Health. 2020;

10. Villani FA, Aiuto R, Paglia L, Re D. Covid-19 and dentistry: Prevention in dental practice, a literature review. Int J Environ Res Public Health. 2020;

11. Brasil. M da S. COVID19 Painel Coronavírus. Coronavirus - Brasil. 2020.

12. Estatística do Estado de São Paulo de Profissionais por Município e População. Conselho Regional de Odontologia de São Paulo (CROSP). 2020.

13. von Elm E, Altman DG, Egger M, Pocock SJ, Gøtzsche PC, Vandenbroucke JP. The strengthening the reporting of observational studies in

epidemiology (STROBE) statement: Guidelines for reporting observational studies. Int J Surg. 2014;

14. Armitage E. Book ReviewGuidelines for Reporting Health Research: A User’s Manual 1st edition, 2014 Moher L, Altman DG, Schulz KF, Simera I, Wager E, eds. Wiley and Blackwell 344 pages. £;29.99 ISBN: 978-0470670446. J Paramed Pract. 2015;

15. Boletim Completo, Casos e óbitos. Fundação Sistema Estadual de Análise de Dados (SEADE). 2020.

16. Cagetti MG, Cairoli JL, Senna A, Campus G. COVID-19 outbreak in North Italy: An overview on dentistry. A questionnaire survey. Int J Environ Res Public Health. 2020;

17. Consolo U, Bellini P, Bencivenni D, Iani C, Checchi V. Epidemiological aspects and psychological reactions to COVID-19 of dental practitioners in the Northern Italy districts of modena and reggio emilia. Int J Environ Res Public Health. 2020;

18. Duruk G, Gümüşboğa ZŞ, Çolak C. Investigation of Turkish dentists’ clinical attitudes and behaviors towards the COVID-19 pandemic: A survey study. Braz Oral Res. 2020;

19. Kamate SK, Sharma S, Thakar S, Srivastava D, Sengupta K, Hadi AJ, et al.

Assessing knowledge, attitudes and practices of dental practitioners regarding the covid-19 pandemic: A multinational study. Dent Med Probl. 2020;

20. Ahmed MA, Jouhar R, Ahmed N, Adnan S, Aftab M, Zafar MS, et al. Fear and practice modifications among dentists to combat novel coronavirus disease (COVID-19) outbreak. Int J Environ Res Public Health. 2020;

21. van der Tas J, Dodson T, Buchbinder D, Fusetti S, Grant M, Leung YY, et al. The Global Impact of COVID-19 on Craniomaxillofacial Surgeons. Craniomaxillofac Trauma Reconstr. 2020;

22. Ghani F. Covid-19 outbreak – immediate and long-term impacts on the dental profession. Pakistan J Med Sci. 2020;

23. González-Olmo MJ, Ortega-Martínez AR, Delgado-Ramos B, Romero-Maroto M, Carrillo-Diaz M. Perceived vulnerability to Coronavirus infection: Impact on dental practice. Braz Oral Res. 2020;

24. Ferneini EM. The Financial Impact of COVID-19 on Our Practice. J Oral Maxillofac Surg. 2020;

25. Ambigapathy S, Rajahram GS, Shamsudin UK, Khoo EM, Cheah WK, Peariasamy KM, et al. How should front-line general practitioners use personal protective equipment (PPE)? Malaysian Family Physician. 2020.

26. Especial COVID-19. UNA-SUS. 2020.

27. COVIDlog. EINSTEIN, H. I. A. 2020.

28. Brownson RC, Eyler AA, Harris JK, Moore JB, Tabak RG. Getting the word out: New approaches for disseminating public health science. J Public Heal Manag Pract. 2018;

29. Scherer CI, Scherer MD dos A. Advances and challenges in oral health after a decade of the "Smiling Brazil" Program. Revista de Saude Publica. 2015.

Figure Legends

Figure 1. Alluvial diagram of workplace (public, private or both) and financial impact (income reduction 0-10%; 10-50% and >50%).

Figure 2. Distribution of the Personal Protective Equipment (PPE) for Infection Control used.

Figure Click here to access/download;Figure;Figures.pdf

Figure 1. Alluvial diagram of workplace (public, private or both) and financial impact (income reduction 0-10%; 10-50% and >50%).

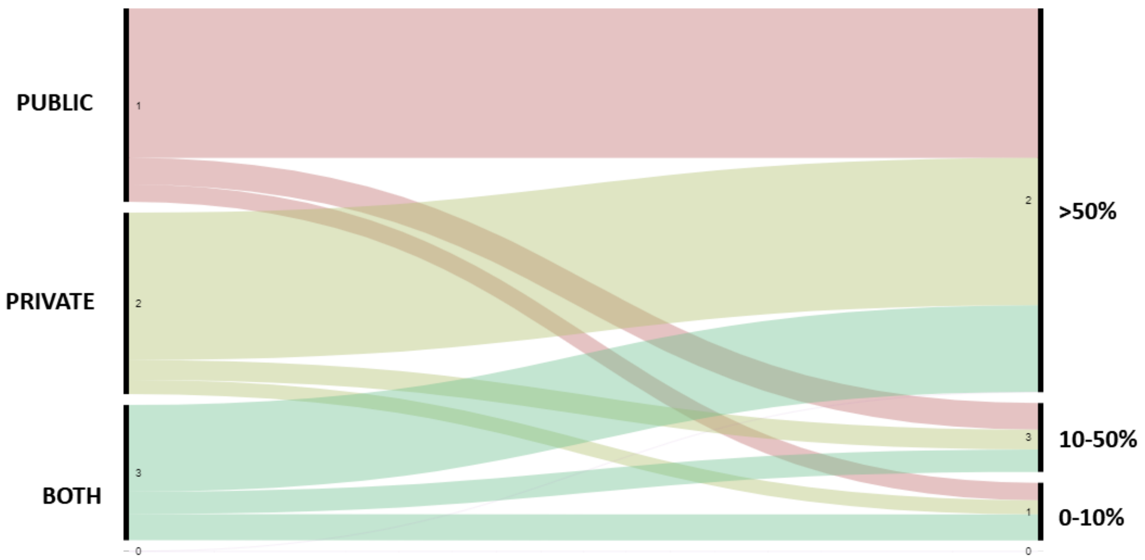

Figure 2. Distribution of the Personal Protective Equipment (PPE) for Infection Control used.

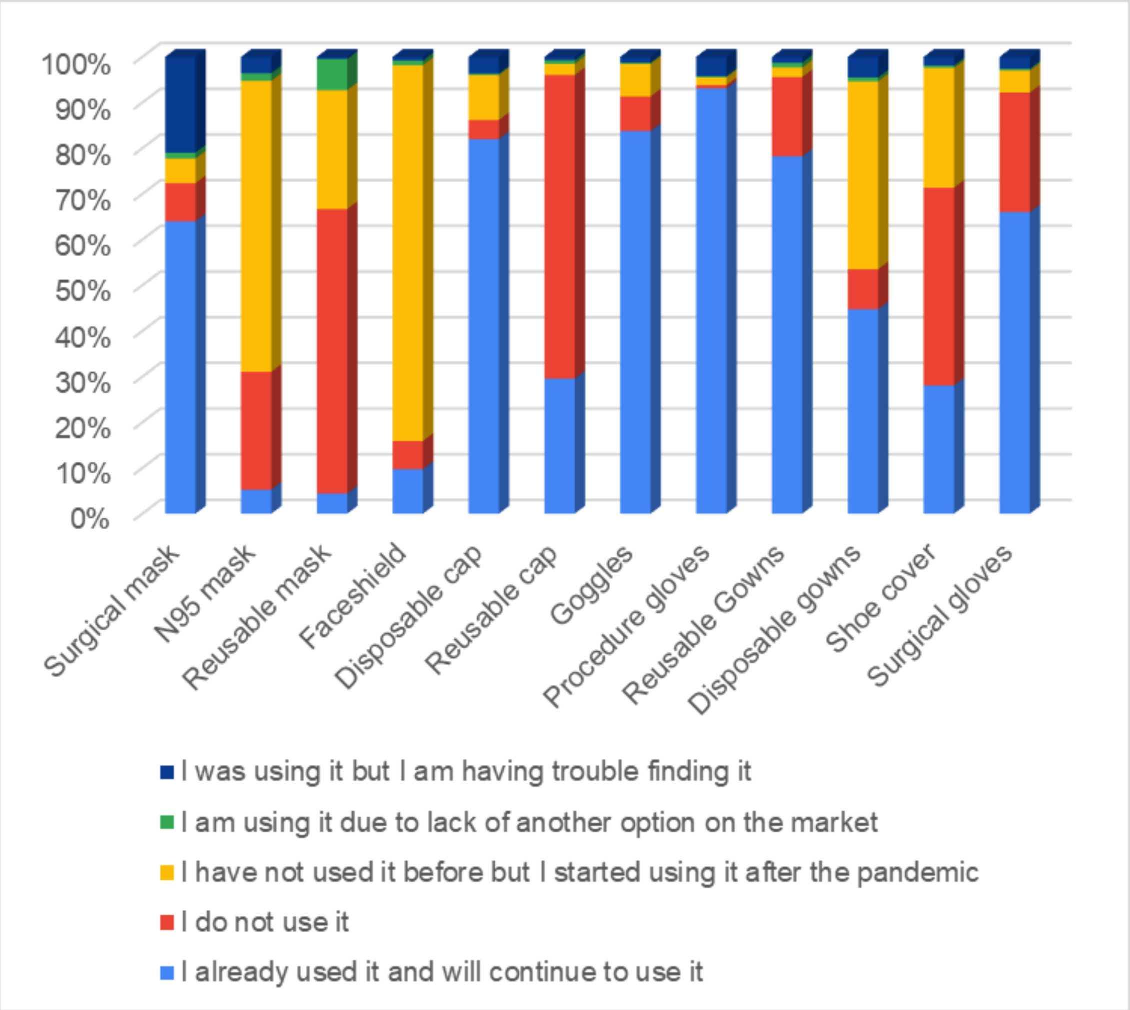

Supplemental Table 1

Click here to access/download
Supporting Information
STROBE check list _PO.docx

Supplemental Table 2

Click here to access/download
Supporting Information
Questionaire_Portuguese_PO.doc

Supplemental Table 3

Click here to access/download
Supporting Information
Questionaire_English_PO.doc
